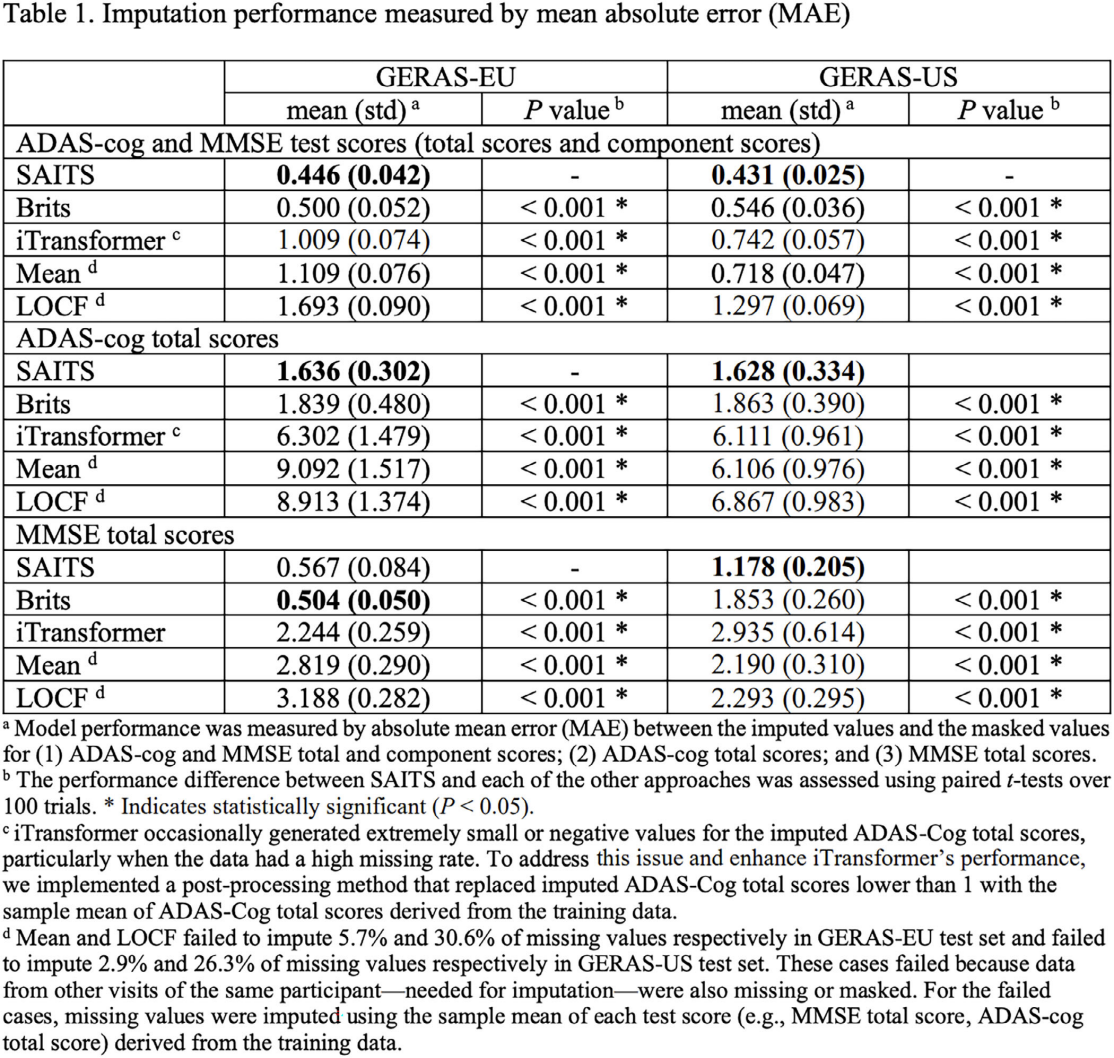# Imputation of Missing Cognitive Assessment Scores in Alzheimer's Disease: A Self‐Attention Based Deep Learning Approach

**DOI:** 10.1002/alz70857_099219

**Published:** 2025-12-24

**Authors:** Bargav Jagatha, Ting Fang Alvin Ang, Rhoda Au, Jinying Chen

**Affiliations:** ^1^ Department of Medicine/Section of Preventive Medicine and Epidemiology, Boston University Chobanian & Avedisian School of Medicine, Boston, MA, USA; ^2^ Department of Compute Science, Graduate School of Arts & Sciences, Boston University, Boston, MA, USA; ^3^ Department of Anatomy & Neurobiology, Boston University Chobanian & Avedisian School of Medicine, Boston, MA, USA; ^4^ Framingham Heart Study, Boston University Chobanian & Avedisian School of Medicine, Boston, MA, USA; ^5^ Slone Epidemiology Center, Boston University Chobanian & Avedisian School of Medicine, Boston, MA, USA; ^6^ Department of Neurology, Boston University Chobanian & Avedisian School of Medicine, Boston, MA, USA; ^7^ Department of Epidemiology, Boston University School of Public Health, Boston, MA, USA; ^8^ Biomedical Genetics, Department of Medicine, Boston University Medical School, Boston, MA, USA; ^9^ Data Science Core, Boston University Chobanian & Avedisian School of Medicine, Boston, MA, USA

## Abstract

**Background:**

Missing data in longitudinal cognitive assessments may occur due to logistical (e.g., scheduling conflicts), personal (e.g., reduced motivation), and health‐related issues (e.g., deteriorated physical or cognitive functions, anxiety, etc.). This issue poses significant challenges for clinical research and patient monitoring. Deep learning‐based imputation approaches, which are agonistic to assumptions of data distribution and covariance structure, outperform traditional methods in imputing longitudinal missing data in other domains. We aim to evaluate their effectiveness in the domain of Alzheimer's Disease (AD), an area that has not been extensively explored.

**Method:**

We developed a deep learning imputation approach using the self‐attention‐based imputation for time series (SAITS) model and de‐identified data accessed on the AD Data Initiative's AD workbench. SAITS imputes missing values in multivariate time series by leveraging self‐attention mechanisms to capture both temporal dependencies and feature correlations. It is trained by jointly optimizing imputation and reconstruction tasks on training data. We trained and evaluated SAITS using the Mini‐Mental State Examination (MMSE) and Alzheimer's Disease Assessment Scale‐Cognitive (ADAS‐cog) test scores from GERAS‐EU and GERAS‐US studies, collected every 6 months over 3 years (7 visits in total). We compared SAITS’ performance with (1) two state‐of‐the‐art deep learning approaches: iTransformer and BRITS and (2) two conventional approaches: mean (averaging data from other visits of the same participant) and last observation carried forward (LOCF). We randomly split the full dataset into 80:20 training:test datasets. Deep learning models were optimized on the training set using 5‐fold cross‐validation and evaluated on the test set with 10% of values randomly masked/removed. Model performance was evaluated using mean absolute error (MAE) across 100 trials.

**Result:**

Data from 1336 GERAS‐EU participants (age: 72.0±11.6, 61.3% female) and 563 GERAS‐US participants (age: 71.0±7.8, 52.7% female) were analyzed. SAITS performed best in most imputation tasks (Tables 1 and 2), achieving 1.636 (GERAS‐EU) and 1.628 (GERAS‐US) MAEs when imputing ADAS‐cog total scores and 0.567 (GERAS‐EU) and 1.178 (GERAS‐US) MAEs when imputing MMSE total scores.

**Conclusion:**

Deep learning techniques demonstrates great potential in imputing missed longitudinal cognitive data in AD. Future work will evaluate their effects on down‐stream tasks such as predicting cognitive decline.